# NEIGHBORHOOD ENVIRONMENTS AND COGNITIVE HEALTH: ADVANCEMENTS IN CONCEPTS AND METHODS

**DOI:** 10.1093/geroni/igae098.1499

**Published:** 2024-12-31

**Authors:** Andrea Rosso, Jana Hirsch

**Affiliations:** Chair; Discussant

## Abstract

Evidence for the importance of residential neighborhood environments for cognitive health in older adults has grown substantially in the past decade. It is important to understand details of these relations, including timing and mechanisms, as interventions at the neighborhood level could have broad public health importance. Here, we present a series of five talks from four studies that aim to advance the field of environmental influences on cognitive health through several conceptual and methodological advancements: inclusion of lifecourse exposures (Cheng, Finlay), consideration of the role of race as a social construct and a modifier of neighborhood exposures (Cheng), incorporation of blood-based biomarkers of Alzheimer’s disease as an outcome (Fan), consideration of spatial and historical context in interpreting results (Moored, Desjardins), use of qualitative interviews to gain insight from people’s lived experiences (Finlay), and use of novel exposures such as cognability (Finlay) and blue space (Moored, Desjardins) as well as upstream factors indicative of structural disadvantage (Cheng, Fan, Desjardin). Together, these talks will challenge the audience to consider the complex ways in which neighborhoods could influence cognitive outcomes and how to best approach analysis and interpretation of such data. Advancing our conceptual understanding of the influence of neighborhoods on cognitive aging over the lifecourse will allow for informed public health interventions. A discussion of next steps will be led by Jana Hirsch. Environmental Gerontology Interest Group Sponsored Symposium

